# Loss of RanBP9 cooperates with p53 deficiency to promote sarcomagenesis

**DOI:** 10.3389/fonc.2026.1743582

**Published:** 2026-09-03

**Authors:** Brianna C. Gonga-Cavé, Gabriel Onea, Xu Wang, Sean P. Cregan, Michael B. Boffa, Patti K. Kiser, Caroline Schild-Poulter

**Affiliations:** 1 Robarts Research Institute, University of Western Ontario, London, ON, Canada; 2 Department of Biochemistry, Schulich School of Medicine and Dentistry, University of Western Ontario, London, ON, Canada; 3 Neuroscience Program, Department of Physiology and Pharmacology, Schulich School of Medicine and Dentistry, University of Western Ontario, London, ON, Canada; 4 Department of Pathology, Schulich School of Medicine and Dentistry, University of Western Ontario, London, ON, Canada; 5 Department of Oncology, Schulich School of Medicine and Dentistry, University of Western Ontario, London, ON, Canada

**Keywords:** CTLH complex, E3 ubiquitin ligase, p53, RanBP9, sarcoma, tumor suppressor proteins

## Abstract

**Introduction:**

Ubiquitination is a major regulatory process that is often dysregulated in carcinogenesis. The C-terminal to LisH (CTLH) complex is a multi-subunit E3 ubiquitin ligase that has been shown to exert tumor-promoting and tumor-suppressing functions. Its core scaffolding subunit, RanBP9, has been implicated in regulating oncogenic signaling, but it remains unclear whether RanBP9 loss promotes tumorigenesis *in vivo*.

**Objectives:**

This study aimed to evaluate the functional consequence of RanBP9 loss, alone or in combination with p53 deficiency, in mice.

**Methods:**

Mice with single or combined germline deletion of *Ranbp9* or *Trp53*, were generated and assessed for *in vivo* tumorigenesis. Tumors were characterized by histology and immunohistochemistry staining. Functional assays in mixed *Ranbp9*/*Trp53* mouse embryonic fibroblasts (MEFs) were performed to evaluate proliferation and survival properties. Global label-free quantification (LFQ) proteomics was performed on MEFs to identify differentially expressed proteins. Genomic alterations of CTLH complex subunits were evaluated in sarcoma patients using the TCGA-SARC dataset.

**Results:**

Biallelic deletion of *Ranbp9* did not induce tumor development. In contrast, double heterozygous *Ranbp9* and *Trp53* (*R9/p53^DHet^*) mice exhibited earlier tumor onset and increased soft tissue sarcoma development compared with *Trp53* heterozygous (*p53^Het^*) mice. Histopathological analysis revealed increased mitotic activity in *R9/p53^DHet^* sarcomas with high Ki-67 and phospho-ERK expression. Functional and proteomic characterization of *R9/p53^DHet^* MEFs demonstrated increased proliferative capacity and changes in proteomic landscape in proteins involved in DNA replication, cell cycle, and metabolism. Finally, TCGA-SARC analysis showed that that low mRNA expression of CTLH catalytic subunit *RMND5B* correlated significantly with reduced overall survival.

**Discussion:**

Loss of RanBP9 induces deregulation of signaling cascades, and combined with p53 deficiency, further promotes sarcoma development in mice. This mouse model provides a valuable platform to further study pathways and candidates regulated by the CTLH complex in sarcoma biology.

## 1 Introduction

Carcinogenesis is driven by the progressive accumulation of multiple genomic alterations that disrupt regulatory networks and promote malignant transformation (1). Ubiquitination is an essential post-translational modification that regulates protein turnover and is frequently perturbed in cancer (2). E3 ubiquitin ligases, which catalyze the covalent attachment of ubiquitin to mainly protein substrates, confer substrate specificity to this process and are often dysregulated in tumors (2–5). Despite their importance, the functions of many E3 ligases in cancer remain poorly defined, and clarifying their roles is necessary to understand how they contribute to tumorigenesis.

The C-terminal to LisH (CTLH) complex is a family of evolutionarily conserved multi-subunit E3 ligase complexes implicated in diverse biological pathways and cancer development (6–9). Approximately 10 proteins are classified as CTLH subunits, many of which contain hallmark LisH, CTLH, and CRA motifs that mediate complex assembly (8). The complex is organized into a scaffold module (RanBP9, GID8, ARMC8α/β), a catalytic heterodimeric RING module (MAEA, RMND5A/B), and interchangeable substrate receptors (GID4, WDR26, MKLN1) (10–15). Structural studies have shown that the CTLH complex adopts distinct conformations—ranging from a clamp-like structure to high-order supramolecular states—that enable fine-tuned substrate recognition (10, 11). RanBP9, a core scaffolding subunit, is essential for maintaining CTLH complex architecture by regulating oligomerization states and recruiting substrate receptors (11, 16, 17). Incorporation of different substrate receptors enables the CTLH complex to recognize and target substrates across multiple cellular pathways including cholesterol biosynthesis, drug metabolism, base excision repair, nucleotide synthesis, and maternal-to-zygotic transition (12, 13, 17–21).

Emerging evidence indicates that the CTLH complex can exert both tumor-promoting and tumor-suppressing properties. Elevated expression of CTLH subunits, including *RANBP9*, has been reported in breast, ovarian, colorectal, and lung cancers (7, 22–29). In contrast, loss of RanBP9 has been associated with enhanced proliferation, clonogenic growth, and xenograft tumor formation, while other studies have identified a pro-apoptotic role for RanBP9 and functions that mediate suppression of cell adhesion (30–35). RanBP9 has also been reported to inhibit cell migration and chemoresistance in gastric tumors (36). Mechanistic studies further indicate that the CTLH complex regulates cell survival, proliferative and metabolic signaling pathways relevant to cancer development (19, 32, 37–39). For instance, the CTLH complex was shown to repress oncogenic signaling pathways, such as the Wnt/β-catenin and MAPK/ERK signaling pathways (32, 38, 40, 41). Recent studies have also demonstrated that the CTLH complex activity is induced in conditions of mTOR inhibition to degrade targets of the mevalonate pathway and the pyrimidine salvage pathway to enable anti-proliferative effects (12, 17, 20). Overall, loss of RanBP9 often enhances proliferative and tumorigenic phenotypes, raising the possibility that it functions as a tumor suppressor *in vivo*. Whether altered CTLH abundance is sufficient to initiate tumorigenesis in mice remains unknown.

Here, we investigated whether *Ranbp9* loss initiates tumorigenesis in a genetic mouse model. We show that *Ranbp9* deficiency altered transcriptional programs associated with oncogenesis but was insufficient to induce tumor development. To determine whether *Ranbp9* loss could accelerate tumor formation in an oncogenic mouse model, we assessed the cooperativity of *Ranbp9* loss in the context of *Trp53* deficiency by breeding mixed *Ranbp9*/*Trp53*-deficient mice. Strikingly, co-deletion of *Ranbp9* and *Trp53* accelerated tumor onset and promoted sarcoma development. These findings provide *in vivo* evidence that CTLH complex disruption can cooperate with *Trp53* deficiency to promote carcinogenesis, supporting a tumor-suppressive function for this E3 ligase in sarcoma development.

## 2 Materials and methods

### 2.1 Generation of mice

*Ranbp9* heterozygous (*R9*^Het^) mice carrying a targeted null mutation for the *Ranbp9* gene (C57BL/6^CR^-*Ranbp9*™/+/Tes) were generously gifted by the laboratory of Lino Tessarolo (43). Due to severe neonatal lethality associated with *Ranbp9*-null mice (43, 44), female C57BL/6^CR^-*Ranbp9*™/+/Tes mice were backcrossed to B6129SF1/J hybrid males (Stock #101043, The Jackson Laboratory) for 5 generations. *R9*^Het^ (B6; 129S) offspring were intercrossed to produce homozygous *Ranbp*9 knockout (*R9^KO^*) mice and control wild-type (WT) littermates. As previously reported (43), *Ranbp9-null* mice (males and females) are sterile. To generate double *Ranbp9* and *Trp53* heterozygous (*R9/p53^DHet^*) and double homozygous *Ranbp9*/*Trp53* knockout (*R9/p53^DKO^*) mice, a two-step breeding strategy was employed. First, *R9*^Het^ (B6; 129S) mice were crossed with B6.129S2-*Trp53^tm1Tyj^*/+/J (Stock #002101, The Jackson Laboratory) mice. *Trp53^tm1Tyj^* mice carry a targeted null mutation for the *Trp53* gene (45). Eight *R9/p53^DHet^* offspring (3 males and 5 females) were intercrossed to generate experimental *R9/p53^DHet^* and *R9/p53^DKO^* mice as well as control *p53^Het^* and *p53^KO^* littermate siblings, although we sometimes had to use *p53^KO^* non-littermate siblings due to the difficulty associated with obtaining *R9/p53^DKO^* mice. Genotyping for *Ranbp9* and *Trp53* was performed by PCR, with primers and reactions conditions provided in **Supplementary Tables 1**, **2**. All colonies were housed in a temperature-controlled facility (21-23 °C) on a 12 h light/dark cycle. Standard laboratory food and water were available *ad libitum*.

### 2.2 Tumor analysis and collection

Experimental cohorts included both male and female mice, monitored biweekly by bodyweight and body-condition scoring for up to 2 years of age. Mice were euthanized when tumors exceeded 1.5 cm in diameter or when poor body-condition was observed. In accordance with SOP# CLN-352, mice were anesthetized with isoflurane (CP0406V2, Fresenius Kabi) using the drop method. To this end, mice were induced in an 1L incubation chamber containing 5% isoflurane. Anesthetic depth was measured by righting reflex and breathing rate. Once mice reached a deep plane of anesthesia, cervical dislocation was performed as a secondary method of euthanasia. Mice were then perfused with phosphate buffered saline (PBS; Wisent Inc.) prior to tissue collection. Tumor samples were isolated and fixed in 4% paraformaldehyde (PFA; BioShop).

### 2.3 Tissue culture and maintenance

Primary mouse embryonic fibroblast (MEF) cells were isolated from pregnant *R9/p53^DHet^* mice mated with *R9/p53^DHet^* males at 13- or 14-day post-coitum (d.p.c.) in a similar method as described previously (46). Cells were maintained in Dulbecco’s modified Eagle medium (DMEM; Wisent Inc.) supplemented with 10% fetal bovine serum (Wisent Inc.), 2 mM _L_-glutamine (Wisent), 1 mM sodium pyruvate (Wisent Inc.), 0.001% β-mercaptoethanol (Thermo Fisher Scientific), and 1% penicillin-streptomycin (Wisent Inc.). Cells were incubated at 37 °C with 5% CO_2_ in a humidified incubator. All cells were passaged at 80% confluence and low passage (< passage 5) cells were used for all experiments.

### 2.4 RNA-sequencing analysis

RNA was extracted from snap frozen liver tissues of 1-month-old *Ranbp9^KO^* (*R9^KO^*) and wild-type (WT) mice using RNeasy Kit (QIAGEN) kit as per manufacturer’s instructions. Concentration and quality of total RNA samples were quantified by Nanodrop (Thermo Fisher Scientific), Agilent 2100 Bioanalyzer (Agilent Technologies Inc), and RNA 6000 Nano kit (Caliper Life Sciences). All samples were sequenced at the London Regional Genomics Centre (Robarts Research Institute; http://www.lrgc.ca) using the Illumina NextSeq 500 (Illumina Inc.). Samples were then processed using the Vazyme VAHTS Total RNA-seq (H/M/R) Library Prep Kit for Illumina (Vazyme) which includes rRNA reduction. In brief, samples were rRNA depleted, fragmented, cDNA was synthesized, indexed, cleaned up and amplified via PCR. Libraries were then equimolarly pooled into one library and size distribution was assessed on an Agilent High Sensitivity DNA Bioanalyzer chip and quantitated using the Qubit 2.0 Fluorimeter (Thermo Fisher Scientific). The library was sequenced on an Illumina NextSeq 500 as a 76 bp single end run, using one High Output v2 kit (75 cycles). For data analysis, Fastq data files were analyzed using Partek Flow (St. Louis, MO). After importation, data was aligned to the Mus musculus mm10 genome using STAR 2.6.1d and annotated using Ensemble v96_v2. Reads were normalized by adding 0.0001 and CLR (Centered Log Ratio) and fold change and p-values were determined using Flow’s Gene Specific Analysis (GSA) between groups of interest. Filtered gene lists were then analyzed for enriched Gene Ontology (GO) biological terms and Reactome pathway terms.

### 2.5 Immunohistochemistry and image acquisition

Fixed tumor tissues were processed into paraffin as previously described (47, 48). Five-micron sections were cut, followed by deparaffinization and rehydration using xylene and ethanol. Sections were stained with Hematoxylin and Eosin (H&E) using an autostainer. H&E-stained sections were blindly assessed by a pathologist (Dr. Patti Kiser) to classify tumors and evaluate tumor burden using a modified histopathological scoring system adapted from the Fédération Nationale des Centres de Lutte Contre Le Cancer (FNCLCC) grading criteria. Images were acquired using a MICA Widefield Automated Microscope (Leica Microsystems) using transmitted light at 10× and 63× magnification.

For Ki-67 analysis, sarcoma sections were stained with anti-Ki-67 antibody (1:1000; MA5-14520, Thermo Fisher Scientific), followed by Alexa Fluor^®^ 647 AffiniPure^®^ Goat Anti-Rabbit IgG (H+L) secondary antibody (1:1000; 111-605-003, Jackson Immuno). Nuclei were counterstained with DAPI (1:3000; D9542, Sigma). Images were acquired using a Stellaris 5 confocal microscope (Leica Microsystems) and taken at 40 x magnification with a Z-step size of 0.5 µm. Laser power was set to 1.18% (407 nm, gain 15%) and 2% (667 nm, gain 11%) and kept constant across all images. Images were processed using Ricard lucy deconvolution (15 iterations) followed by maximum intensity projection.

For c-Jun and phospho-ERK staining, sarcoma sections were stained with anti-c-Jun (60A8) antibody (1:600; 9165, Cell Signaling Technology) or anti-phospho-p44/42 MAPK (Erk1/2) (Thr202/Tyr204) antibody (1:500; 9101, Cell Signaling Technology), followed by DAB detection. Nuclei were counterstained with hematoxylin. Images were acquired using a MICA Microscope using transmitted light at 20× magnification, with exposure set to 40 ms and gain set to 1%. All image acquisition settings were kept constant.

Quantification of Ki-67, c-Jun, and phospho-ERK expression was performed with pixel and object classification using a machine-learning workflow in ilastik (version 1.4.1) (49).

### 2.6 Western blotting

Cells were collected by scraping and pelleted at 300g for 5 min at 4 °C. Pellets were lysed in RIPA buffer (150 mM NaCl, 50 mM Tris-HCl (pH 8), 1% NP-40, 0.5% Sodium Deoxycholate, 0.1% Sodium Dodecyl Sulfate (SDS)) supplemented with protease inhibitors (0.2 mM PMSF, 1 mM DTT, 1 g ml^−1^ leupeptin, 10 g ml^−1^ aprotinin, 1 g ml^−1^ pepstatin (inhibitors obtained from BioShop). Protein concentrations were determined using Pierce™ 660nm Protein Assay Reagent (22660, Thermo Fisher Scientific) supplemented with Ionic Detergent Compatibility Reagents (22663, Thermo Fisher Scientific) according to the manufacturer’s recommendations.

Protein samples (25 µg per sample) were boiled for 10 min at 95 °C, separated by 8% or 10% SDS-PAGE gels, and transferred to Immobilon-FL PVDF membrane (IPFL00005, Sigma) using the semi-dry transfer method. Blots were blocked in fish gelatin (0.5%; Sigma) in TBST (0.05% Tween; BioShop) for 1 h at room temperature. Blots were incubated overnight with primary antibodies diluted in TBST containing 0.5% fish gelatin. After 16 h, blots were washed with TBST for 25 min at room temperature and incubated with secondary antibodies diluted in TBST buffer containing 0.5% fish gelatin and 0.02% SDS. Western blot antibodies details are reported in **Supplementary Table 3**. Blots were washed for 25 min with TBST buffer in the dark at room temperature. Blots were imaged using ChemiDoc (BioRad). Band intensities were quantified using ImageLab (BioRad).

### 2.7 Growth curve assay

Cells were seeded in triplicate at 2 × 10^3^ cells per well in a 96-well tissue culture plates. Cell density was measured at indicated time points using the Cell Counting Kit 8 (CCK-8; Ab228554, Abcam) according to the manufacture’s protocol. Briefly, 10µL of WST-8 solution was added to each well containing 100 µL of medium and incubated for 2.5 h in a humidified incubator (37 °C, 5% CO_2_). Absorbance was measured at 450 nm using a microplate reader. Values were normalized to blanks and quantified using a standard curve according to the manufacturer’s recommendations.

### 2.8 DNA damage treatments

Cells were exposed to 4 Gy ionizing radiation (CellRad™, Precision X-ray Inc) or treated with 10 µM cisplatin (232120, Sigma) for 24 h in standard growth conditions (37 °C, 5% CO_2_) and allowed to recover as indicated in the figures. Cisplatin was freshly prepared in PBS immediately prior to use. For olaparib treatment (SML3705, Sigma), cells were treated with varying olaparib concentrations for 72 h. Control samples received PBS or DMSO.

### 2.9 Cell viability assay

Cells were seeded in triplicate in a 96-well plate and subjected to ionizing radiation, cisplatin, or olaparib. Cell viability was measured at indicated time points using the CCK-8 assay (as described in Growth curve assay), quantified by normalizing absorbance values. For olaparib treatment, inhibitory concentrations (IC_50_) values for each genotype group tested was determined by non-linear regression of log transformed olaparib concentration against normalized response.

### 2.10 Colony formation assay

Cells were seeded at single-cell density in a 60 mm dishes coasted with gelatin B (0.2%; Sigma) in triplicates. After 16 h, cells were irradiated and cultured for 15 days. Colonies were washed with PBS, fixed, and stained with crystal violet solution (0.5% in 20% methanol) for 15 minutes, then extensively washed with PBS. Plates were imaged using ChemiDoc and colonies containing ≥ 50 cells were counted manually.

### 2.11 Global mass spectrometry sample preparation

WT (*n = 4*), *R9^He^*^t^ (*n = 3*), *p53^Het^ (n* = 8*)*, and *R9/p53^DHet^* (*n* = 9) MEFs (from at least 3 different litters) were seeded at 1 × 10^6^ cells per 10 cm plates and were collected the following day once cells reached 80% confluency. Only early passage cells (passage 2 and 3) were used. Sample processing was performed in the Biomolecular Core (BioCORE) Facility at Western University Schulich School of Medicine and Dentistry, London, Canada, using the single-pot, solid-phase-enhanced sample preparation strategy (SP3) similar to previously described methods (50, 51). Briefly, cells was homogenized in 1% sodium deoxycholate (SDC), 0.1 M Tris(hydroxymethyl)aminomethane pH 8, and sonicated by probe-tip using a Sonic Dismembrator Model 100 (FisherScientific) with a 5 s ON 30 s OFF cycle and amplitude setting of 2. Protein extracts were centrifuged at 16,400 × g for 15 min at 20 °C, quantified by Pierce BCA Protein Assay Reagent (ThermoFisher Scientific), reduced with 20 mM Tris(2-carboxyethyl)phosphine (TCEP; ThermoFisher Scientific), alkylated with 40 mM iodoacetamide (Sigma) at 60 °C, then mixed 1:10 with SP3 beads (Cytiva), and precipitated from an equal volume of 100% ethanol. Beads were washed with 80% ethanol, resuspended in 50 mM ammonium bicarbonate pH 8 (Fisher Scientific), and digested 1:50 w/w with lysyl endopeptidase (LysC; FUJIFILM Wako Chemicals) for 4 h at 37 °C shaking 1400 rpm, followed by addition of trypsin (MS grade; ThermoFisher Scientific) at 1:50 w/w overnight as above. Sample peptide supernatants were collected, then beads washed with 2% acetonitrile (ACN) containing 0.5% trifluoroacetic acid (TFA), washes pooled to acidify peptide digests to ~0.5% TFA. Peptides were desalted using Oasis HLB 1cc (10 mg; Waters) solid-phase extraction cartridges, eluates evaporated to dryness by speed vacuum concentration, and resultant peptides resuspended in 2% ACN, 0.1% TFA shaking 1400 rpm 5 min, then ultrasonic bath for 20 min. Peptide samples were centrifuged at 21,000 x g for 7 min at room temperature, quantified by microBCA assay (ThermoFisher Scientific), then transferred to Thermo SureSTART (ThermoFisher Scientific; 6PK1655) autosampler vials for injection.

### 2.12 Liquid chromatography mass spectrometry

Liquid chromatography mass spectrometry (LC-MS/MS) was performed in the Biomolecular Core (BioCORE) Facility at Western University Schulich School of Medicine and Dentistry, London, Canada. Peptide samples were injected onto a Vanquish Neo UHPLC System coupled to an Orbitrap Eclipse Tribrid quadrupole-ion trap-Orbitrap mass spectrometer (ThermoFisher Scientific). Samples were loaded onto a PepMap Neo Trap Cartridge, C18, 100Å, 5 μm, 300 μm × 5 mm (ThermoFisher Scientific) and resolved on a nanoEase Peptide BEH C18 Column, 130Å, 1.7 μm, 75 μm × 250 mm (Waters) at 55 °C and a flow rate of 300 nL min-1 using a nonlinear gradient (2.5-8.8% B, 1 min; 8.8-32% B, 46 min; 32-50% B, 5 min; 50-99% B, 1 min) of H2O/0.1% formic acid (FA) (Mobile Phase A) and 80/20 ACN/H2O with 0.1% FA (Mobile Phase B).

The Orbitrap Eclipse Tribrid mass spectrometer was controlled by Xcalibur software (version 4.7; Thermo Fisher Scientific). Data were acquired in data-independent acquisition mode (DIA) similar to previously described methods (52, 53). Briefly, staggered, overlapping 8 m/z precursor isolation windows were collected over a mass range of 390-1010. Survey scans (MS1) were acquired in centroid profile at a resolution of 60,000 with normalized AGC (automatic gain control) target set to 100% (4e5) and maximum injection time set to 60 ms. Tandem MS scans (MS2) were acquired in centroid profile over a mass range of 145–1450 m/z and fragmented using HCD with a NCE (normalized collision energy) set to 30%. Fragment ions were acquired at a resolution of 15,000 with normalized AGC (automatic gain control) target set to 660% (3.3e5), maximum injection time set to 22 ms, loop count of 75, and default charge of 3. All liquid chromatography (LC) and mass spectrometry (MS) solvents used were of Optima LC/MS grade from Fisher Scientific.

### 2.13 Proteomic data processing

All raw LC-MS/MS data files were processed with PEAKS Studio (version 12.0; Bioinformatic Solutions Inc.) using the PEAKS proteome workflow search engine. Precursor and fragment mass error tolerance was set to 10.0 ppm and 0.02 Da, respectively. The match-between-runs (MBR) setting was applied. Enzyme specificity was set to trypsin, with up to two maximum missed cleavages. Carbamidomethylation of cysteine (+57.0215 Da) was set as fixed modification. Deamidation of asparagine and glutamine (+0.984 Da) and oxidation of methionine (+15.9949 Da) were set as variable modifications. Peptides with a maximum of 4 variable PTMs were retained. Datasets were searched against the *Mus musculus* canonical database (UP000000589; downloaded 2025-09-22) with the following parameters: peptide length set to 4-30, precursor m/z set to 390-1010, fragment m/z set to 145-1450, and charge set to 1-6. Peptides with an average area ≥ 20000.0, quality ≥ 20.0, FDR < 1% and charge 1–6 were retained. Data were quantified by label-free quantification (LFQ) using identification directed quantification. Top 3 algorithmic summarization methods were applied. Using the same workflow, datasets were analyzed in two analysis batches: (1) comparing the proteome of all 4 genotypes with WT replicate 1 (Sample ID: WT3-4) as the base sample and (2) comparison between *R9/p53^DHet^* and *p53^Het^* proteomes with *p53^Het^* replicate 1 (Sample ID: p531-3) as the base sample.

### 2.14 Functional proteomic analysis

For downstream analysis, only proteins with two or more peptides, a minimum of one unique peptide, and assigned to a unique protein group were retained. Raw intensity values were log_2_-transformed. For principal component analysis (PCA), proteins with an absolute fold change of at least 1.2 and a *p*-value less than 0.05 (determined by ANOVA) were retained. For differentially expressed analysis, variance stabilizing was applied for normalization, and imputation was performed using K-nearest neighbor (KNN) for missing values. The Linear Models for Microarray Data (limma) R package was used to calculate differential expression between either *R9^Het^*, *p53^Het^*, or *R9/p53^DHet^* proteomes against WT proteomes or *R9/p53^DHet^* proteome against *p53^Het^* proteome. Differential expressed proteins (DEP) with an absolute fold change greater than 1.2 and a *p*-value lesser than 0.05 were considered as significant. DEPs were visualized by volcano plot. For functional enrichment analysis, upregulated DEPs were searched against the Reactome database for biological pathway network. Pathways with an FDR less than 0.1 and an entities p-value less than 0.05 were considered as significant. Pathways categorized under human disease level were omitted. The remaining pathways categorized as a level 2 or 3 were visualized by dot plot. Median-centered LFQ intensities were calculated using log_2_ intensities subtracted by sample median centered log_2_ intensities.

### 2.15 Human cancer bioinformatics analysis

Gene expression and genomic alteration analyses were performed using publicly available datasets accessed through cBioportal (54, 55). For mutual exclusivity analysis, classic tumor suppressor genes were identified using the TSGene database (56), and exclusivity with *RANBP9* was assessed in the TCGA PanCancer Atlas cohort using cBioPortal. To this end, Log_2_ odds ratios were used to quantify the strength and direction between two genetic events, and a two-sided Fisher Exact test was used to determine statistical significance. For evaluation of the status of CTLH complex genes in sarcomas, the TCGA-SARC PanCancer Atlas dataset was queried using default parameters in cBioPortal. Mutation and alteration frequencies were obtained from the “Plots” and “Mutations” tabs, and full CNA data were downloaded for validation. Expression values were reported as log_2_(TPM + 1), and copy-number alterations were determined using GISTIC2.0. For Cox proportional hazard regressions, the TCGA-SARC PanCancer dataset was downloaded from cBioPortal and fitted to a cox proportional hazard model. Expression for each CTLH subunit gene was reported as a continuous variable with *p*-value adjusted for age, sex, and histology. Kaplan-Meier survival analyses were performed to estimate survival probabilities across CTLH subunit mRNA expression grouped by quartiles.

### 2.16 Statistical analysis

All statistical analyses were performed in GraphPad Prism (version 10.1.1) or Rstudio (version 4.4.3). The number of independent replicates, statistical tests, and associated metrics are specified in each figure legend. Results were considered significant at *p* < 0.05(*), *p* < 0.01(**), *p* < 0.001(***), and *p* < 0.0001(****).

### 2.17 Data visualization

Data visualization was performed using GraphPad Prism and Rstudio. Schematics were created with BioRender.

## 3 Results

### 3.1 *Ranbp9* knockout mice do not develop spontaneous tumors

**Figure 1:**
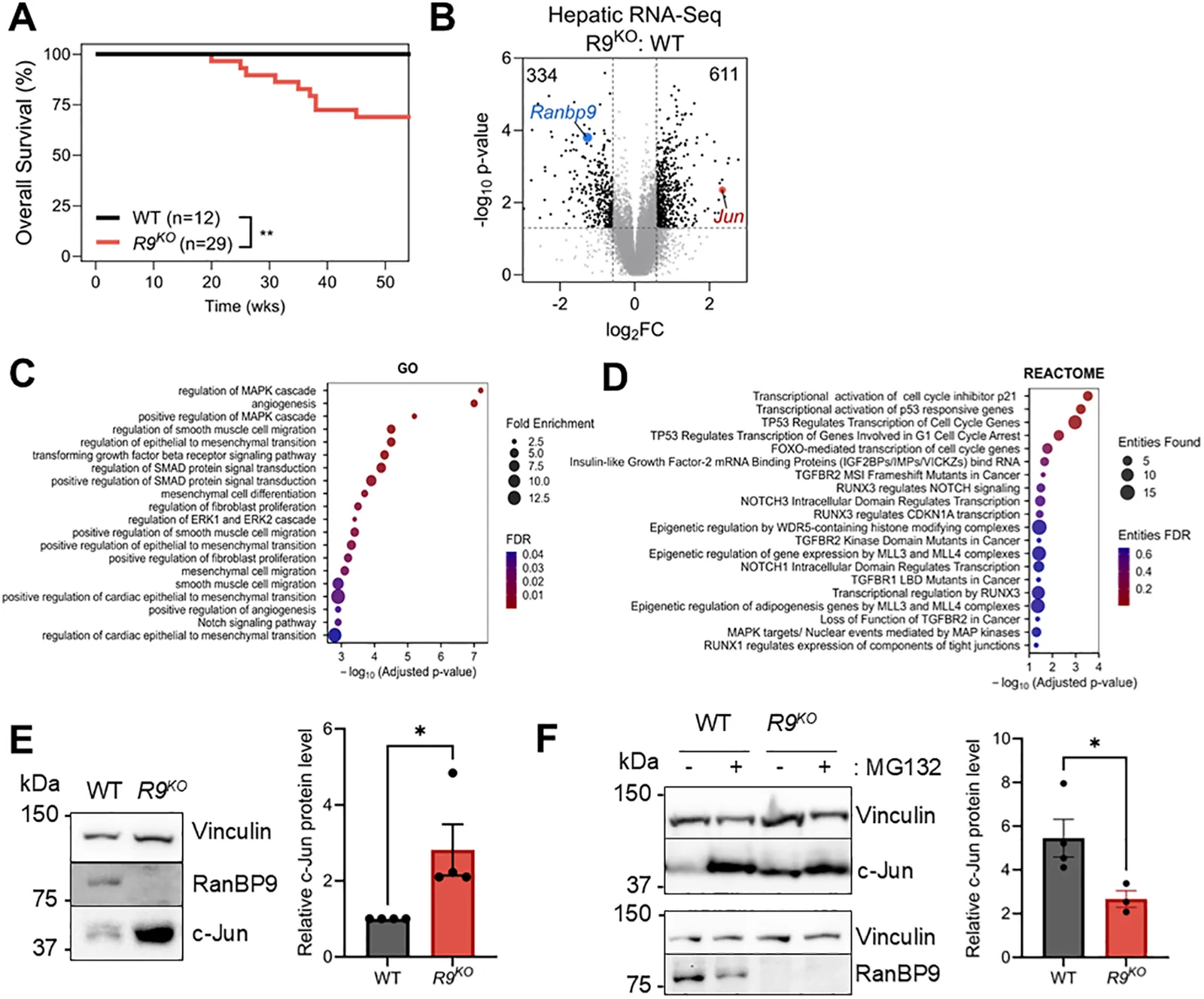
*Ranbp9* knockout promotes oncogenic signaling in mice. **(A)** Kaplan-Meier overall survival of wild-type (WT) and *Ranbp9 knockout (R9^KO^)* mice. Statistical significance was determined by log-rank test. **p < 0.01. **(B)** Volcano plot of differentially expressed genes (DEGs) in livers from 1-month old R9^KO^ (*n* = 5) and WT (*n* = 7) mice. **(C, D)** Gene ontology (GO) and Reactome pathway enrichment of upregulated DEGs from R9^KO^ livers. Adjusted *p*-values were calculated using the Benjamini-Hochberg method. **(E)** Western blot analysis of c-Jun protein levels in WT and *R9^KO^* MEFs. Vinculin served as a loading control. Quantification (right) shows mean ± SEM relative to WT (*n* = 4). Statistical significance was determined by unpaired t-test. *p < 0.05. **(F)** WT and *R9^KO^* MEFs were treated with MG132 for 16 h prior to western blot analysis. Negative controls received DMSO. Vinculin served as a loading control. Quantification (right) shows mean ± SEM relative to WT (*n* > 3). Statistical significance was determined by unpaired t-test. *p < 0.05. RanBP9 was assessed on a different western blot with the same samples.

To investigate the *in vivo* tumor suppressive role of the CTLH complex, we first evaluated single homozygous *Ranbp9* knockout (*R9^KO^*) mice for the development of spontaneous tumors. We used *Ranbp9* loss as a proxy for CTLH E3 ligase impairment as RanBP9 deficiency disrupts CTLH complex formation (11, 15–17). We observed no tumor development in *R9^KO^* mice over a 2-year observational period. Instead, *R9^KO^* mice were susceptible to developmental comorbidities including severe dermatitis and malocclusions (43), leading to humane endpoints. Consequently, *R9^KO^* mice showed a significant reduction in overall survival compared to WT littermate controls (**Figure 1A**). Post-mortem analysis revealed *R9^KO^* mouse livers were pale relative to WT controls (**Supplementary Figure 1**). β-galactosidase staining revealed enhanced staining in *R9^KO^* liver, suggesting a larger population of senescent cells in these livers.

To characterize the molecular consequences of *Ranbp9* loss, we assessed the hepatic transcriptome of *R9^KO^* mice by RNA sequencing. Hierarchical clustering demonstrated a clear segregation between *R9^KO^* and WT liver tissue (**Supplementary Figure 2**). We identified 945 differentially expressed genes (DEGs) in liver of *R9^KO^* compared to WT mice (with an absolute fold change of 1.5 and *p-value* less than 0.05; **Supplementary Dataset 1**). As anticipated, *Ranbp9* was among the top significantly downregulated genes (-2.4-fold, *p* = 1.57 ×10^-4^) compared to WT controls (**Figure 1B**). Proto-oncogene *Jun* (encoding the activator protein-1 transcription factor c-Jun) was significantly upregulated (5.1-fold, *p* = 4.47×10^-3^). Pathway analysis of upregulated DEGs showed an enrichment in differentiation processes, cellular migration, and several proliferation cascades including MAPK/ERK pathway, which is known to be negatively regulated by the CTLH complex via c-Raf in HeLa cells (**Figure 1C, D**; **Supplementary Dataset 2**) (32, 41). Because c-Jun was recurrently identified in previous transcriptomic and proteomic screens involving members of the CTLH complex (13, 57), we investigated this regulation further. We confirmed that knockout of RanBP9 significantly increased c-Jun protein levels in MEFs (2.5-fold, *p* = 3.62×10^-2^; **Figure 1E**) suggesting that this regulation is preserved in mesenchymal cells. Furthermore, *R9^KO^* MEFs treated with a proteasomal inhibitor, MG132, displayed a reduced stabilization of c-Jun relative to WT controls (**Figure 1F**), indicating loss of RanBP9 affects c-Jun degradation. Immunoblot analysis of c-Jun protein expression across CTLH-deficient HeLa cell lines also revealed elevated c-Jun protein levels (**Supplementary Figures 3A–E**). Lastly, cycloheximide (CHX) chase experiments confirmed increased c-Jun protein stability in *MAEA^KO^* cells compared to WT cells, indicating c-Jun degradation is dependent on CTLH complex activity (**Supplementary Figure 3F**). Overall, these analyses suggest the CTLH complex restricts c-Jun expression both at the transcriptional and post-translational levels across different cell types.

### 3.2 Heterozygous deletion of *Ranbp9* and *Trp53* in mice accelerates tumor onset and promotes development of soft tissue sarcomas

**Figure 2:**
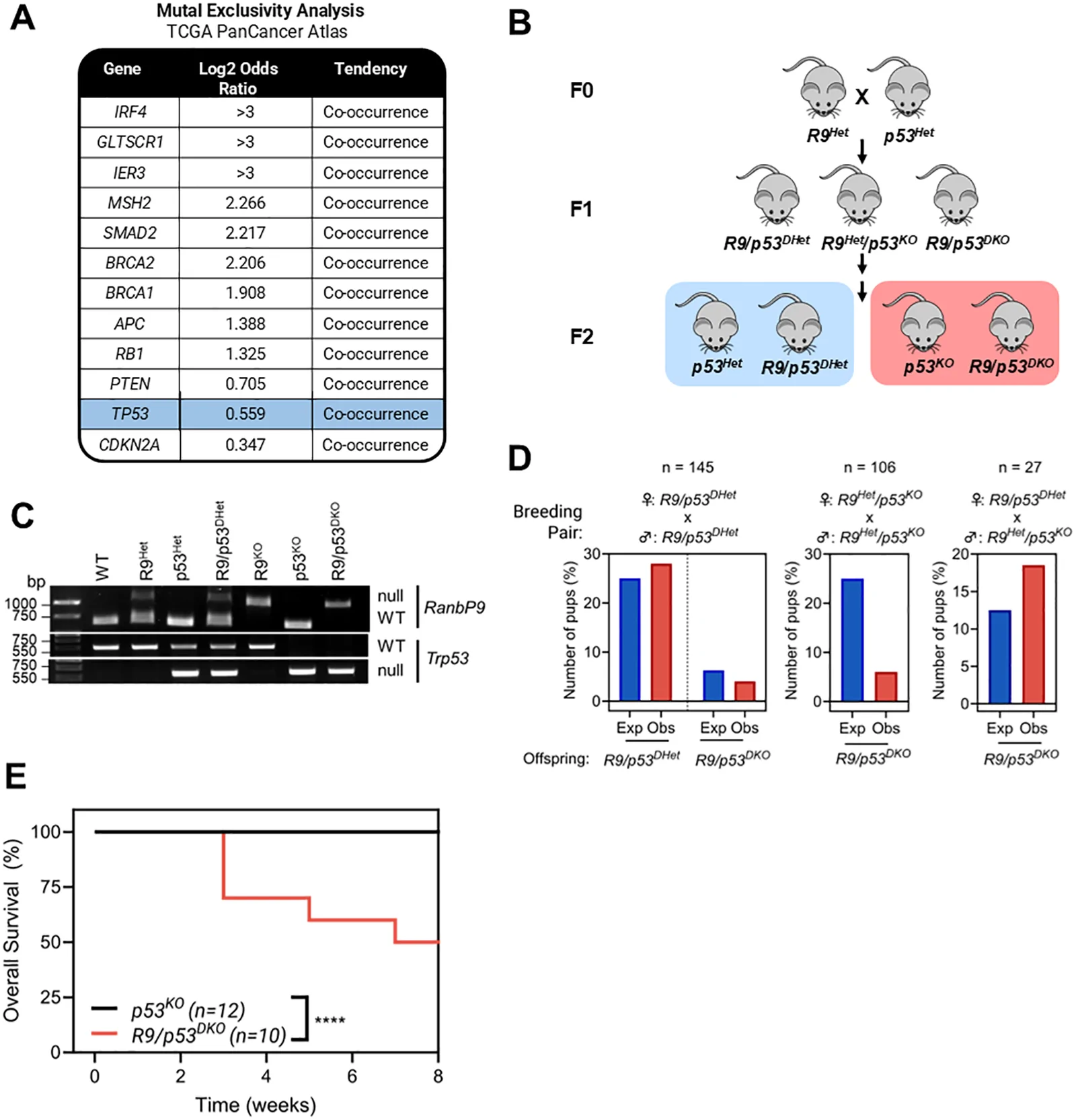
Generation of mixed *Ranbp9/Trp53* deficient mice. **(A)** Mutual exclusivity analysis of *RANBP9* and selected tumor suppressor genes across human cancers from TCGA PanCancer Atlas. **(B)** Breeding strategy used to generate double heterozygous (*R9/p53^DHet^*), double knockout (*R9/p53^DKO^*), and control mice. **(C)** Genotype PCR confirming *Ranbp9* and *Trp53* status in mice. **(D)** Mendelian distributions of *R9/p53^DHet^* and *R9/p53^DKO^* offspring. **(E)** Kaplan-Meier overall survival of *R9/p53^DKO^* and *p53^KO^* mice during the first 8 weeks of life. Statistical significance was determined by log-rank test. ****p < 0.0001.

Tumorigenesis often requires multiple genetic alterations. We therefore hypothesized that the loss of RanBP9 may enhance malignant transformation following the loss of a canonical tumor suppressor gene (TSG). First, we performed a mutual exclusivity analysis to identify TSGs that are frequently co-altered with *RANBP9*. TCGA Pan-Cancer analysis identified 73 of 76 key TSGs harbored genetic alterations that co-occur with *RANBP9* alterations (**Figure 2A**). We observed that mutations in *TP53* co-occur with those in *RANBP9*. Based on this and given that p53 function is inactivated in most human cancers, we chose to generate mice deficient in both *Ranbp9* and *Trp53* to investigate whether loss of RanBP9 potentiates the tumorigenic effects of p53 deficiency. Double heterozygous (*R9/p53^DHet^*) and double knockout (*R9/p53^DKO^*) mice were generated through successive intercrossing and genotyped for identity (**Figure 2B, C**). *R9/p53^DHet^* mice displayed expected Mendelian ratio (**Figure 2D**). In contrast, *R9/p53^DKO^* mice were difficult to generate as these mice deviated from the expected Mendelian ratio using *R9/p53^DHet^* breeders, consistent with *Ranbp9*-null mice. Crossing *R9/p53^DHet^* females with *R9^Het^/p53^KO^* males increased the frequency of *R9/p53^DKO^* mice, although the sample size remained limited. As expected, *R9/p53^DKO^* mice exhibited multiple health issues (dermatitis and malocclusions) due to *Ranbp9* homozygosity, leading to early euthanasia (**Figure 2E**). Difficulties with generating *R9/p53^DKO^* mice resulted in a limited cohort size for subsequent observational analysis.

**Figure 3:**
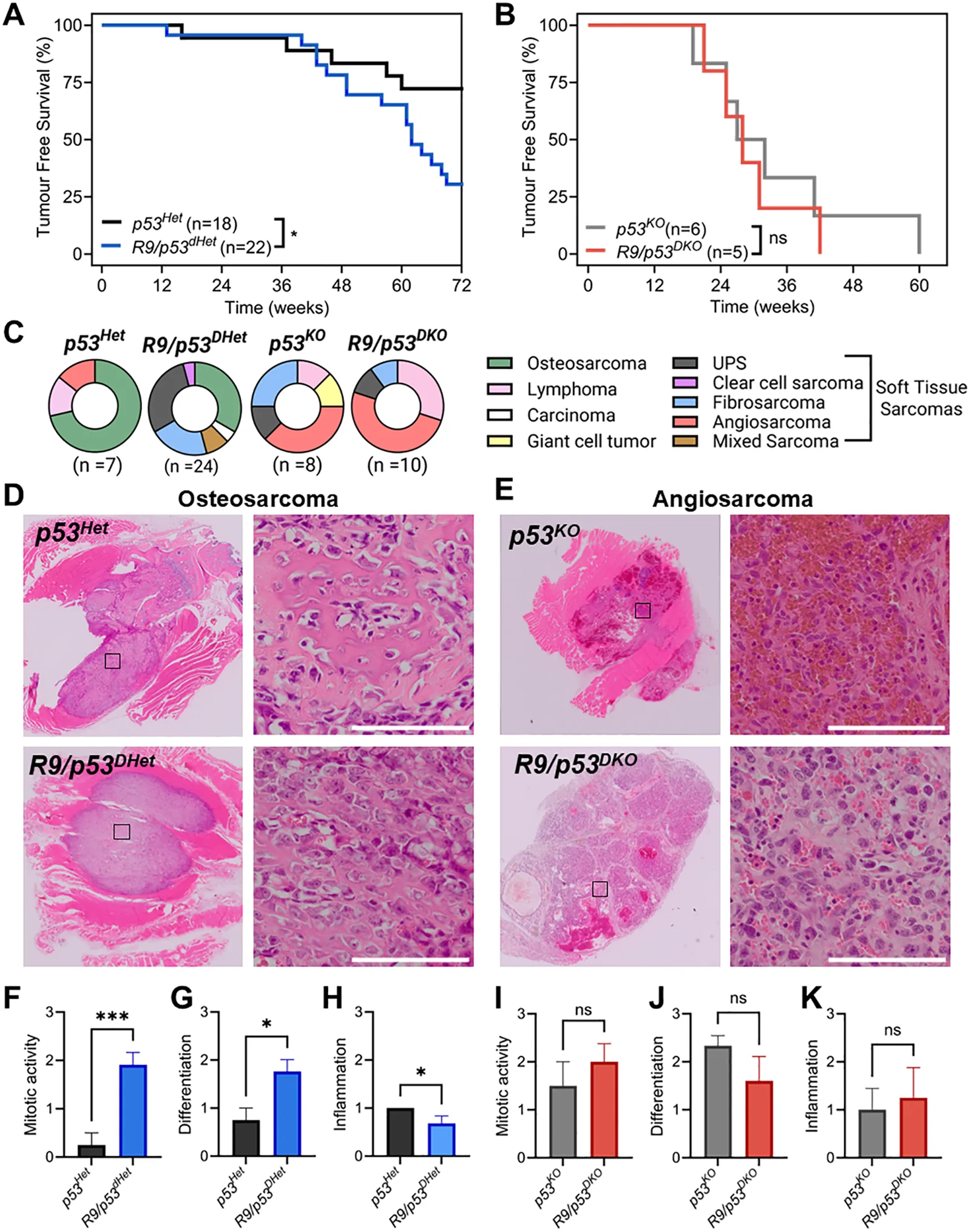
*Ranbp9* loss cooperates with *Trp53* deficiency to promote sarcoma development. **(A, B)** Kaplan-Meier tumor-free survival of *p53^Het^*, *R9/p53^DHet^*, *p53^KO^*, and *R9/p53^DKO^* mice. Statistical significance was determined by log-rank test. *p < 0.05; ns = non-significant. **(C)** Identification of tumors developed in mice by a pathologist’s blind assessment. UPS = Undifferentiated Pleomorphic Sarcoma. **(D, E)** Hematoxylin and eosin (H&E) staining of osteosarcoma and angiosarcoma sections. Scale bar represents 100 µm. **(F–K)** Histological grading of mitotic activity, differentiation, and immune infiltration of sarcomas sections. Quantification of index grade is shown as mean ± SEM. Statistical significance was determined by unpaired t-test with Welch’s correction. *p < 0.05; ***p < 0.001; ns, non-significant.

The loss of p53 in mice results in the spontaneous development of sarcomas and lymphomas at defined time points (45). Mixed *R9/Trp53* mouse cohorts were monitored for the development of spontaneous tumors for over 72 weeks to assess whether RanBP9 could promote earlier tumor development and potentially alter tumor spectrum of tumor types observed in the p53-deficient mouse model. Mice were euthanized upon detection of tumors greater than 1.5 cm in diameter or presentation of cancer symptoms. For Het cohort, Kaplan-Meier analysis revealed that *R9/p53^DHet^* displayed a reduced tumor-free survival compared to *p53^Het^* controls (*p* = 0.0482; **Figure 3A**). *R9/p53^DHet^* had a median survival of 62 weeks and only 30% of mice remained tumor-free. In contrast, approximately 72% of *p53^Het^* mice remained tumor free by 72 weeks. For the KO cohort, the median tumor-free survival was similar, at 28 weeks for *R9/p53^DKO^* and 29.5 weeks for *p53^KO^* mice (*p* = 0.6253; **Figure 3B**). Characteristics and morbidities associated with all mice lines in this study are reported in **Supplementary Table 4**.

Next, we investigated whether *Ranbp9* deletion alters typical tumor subtypes associated with p53-deficient mice. For the heterozygous cohort, *R9/p53^DHet^* mice primarily developed soft tissue sarcoma (STS), with 29% of tumors being undifferentiated pleomorphic sarcomas (UPS), followed by 20% of tumors being fibrosarcoma (**Figure 3C**). *p53^Het^* mice primarily developed osteosarcomas (71%) (**Figure 3D**). Unexpectedly, *R9/p53^DKO^* and *p53^KO^* mice primarily developed angiosarcomas rather than the typical lymphoma-prone phenotype commonly observed in *p53^KO^* mice (45) (**Figure 3E**). To further characterize sarcomas, tumors were scored based on histopathological presentation. Overall, all 4 groups displayed moderately pleomorphic sarcomas with moderate nuclear atypia (**Supplementary Figure 4**). *R9/p53^DHet^* sarcomas displayed significantly increased mitotic activity compared to *p53^Het^* sarcomas (*p* = 0.0007; **Figure 3F**). Additionally, sarcomas from *R9/p53^DHet^* mice were moderately differentiated compared to sarcomas from *p53^Het^* mice (*p* = 0.0161; **Figure 3G**). Lastly, immune infiltration was mild in both *R9/p53^DHet^* and *p53^Het^* sarcomas, with *R9/p53^DHet^* exhibiting a decrease in immune infiltration (*p* = 0.0496; **Figure 3H**). For the KO cohort, there was no significant difference in mitotic activity, differentiation, and immune infiltration between the 2 groups (**Figures 3I–K**).

**Figure 4:**
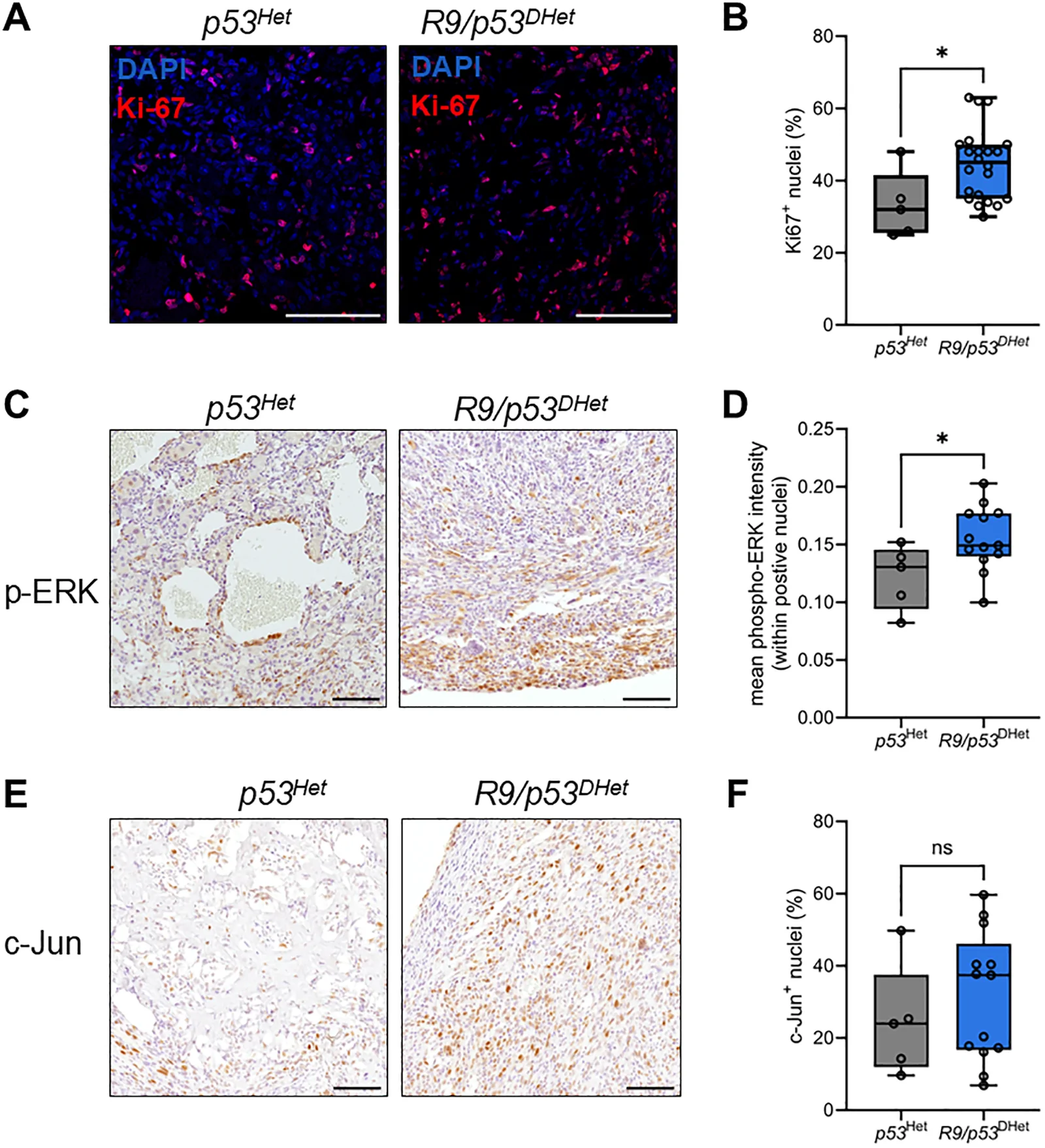
Enhance Ki-67 and phospho-ERK expression in *R9/p53^DHet^* sarcomas. **(A)** Ki-67 staining and nuclear counterstain were performed on sarcoma sections from *R9/p53^DHet^* and *p53^Het^* mice (scale bar represents 100 µm). **(B)** Quantification of Ki-67 positive (Ki67^+^) nuclei in *R9/p53^DHet^* (*n* = 22) and *p53^Het^* (*n* = 5) sarcomas is shown as mean ± SEM. A minimum of 400 nuclei were counted per biological replicate. Statistical significance was determined by unpaired t-test with Welch’s correction. *p < 0.05. **(C)** phospho-ERK (p-ERK) staining and hematoxylin nuclear counterstain were performed on sarcoma sections from *R9/p53^DHet^* and *p53^Het^* mice (scale bar represents 100 µm). **(D)** Quantification of mean p-ERK intensity within positive nuclei *R9/p53^DHet^* (*n* = 13) and p53^Het^ (*n* = 5) sarcomas is shown as mean ± SEM. A minimum of 400 nuclei were counted per biological replicate. Statistical significance was determined by unpaired t-test with Welch’s correction. *p < 0.05. **(E)** C-Jun staining and hematoxylin nuclear counterstain were performed on sarcoma sections from *R9/p53^DHet^* and *p53^Het^* mice (scale bar represents 100 µm). **(F)** Quantification of c-Jun positive (c-Jun^+^) nuclei in *R9/p53^DHet^* (*n* = 13) and *p53^Het^* (*n* = 5) sarcomas is shown as mean ± SEM. A minimum of 400 nuclei were counted per biological replicate. Statistical significance was determined by unpaired t-test with Welch’s correction. ns, non-significant.

Finally, immunohistochemical analysis of Ki-67 expression showed increased Ki-67 expression in *R9/p53^DHet^* sarcomas (*p* < 0.05), consistent increased proliferation (**Figure 4A, B**). Given our earlier observations with aberrant c-Jun stabilization and upregulated ERK signaling due to RanBP9 loss, we reasoned that c-Jun and ERK signaling may contribute to enhanced sarcoma development. Indeed, we observed clear enhanced phospho-ERK intensity in *R9/p53^DHet^* sarcomas relative to *p53^Het^* controls (*p* < 0.05; **Figure 4C, D**). C-Jun staining of the adjacent sections were heterogenous in *R9/p53^DHet^* cohort with some samples expressing high levels of c-Jun and others expressing low levels of c-Jun (**Figure 4E**). A trend in global increase in c-Jun intensity was also detected but did not reach significance (**Figure 4F**). These findings suggest that loss of *RanBP9* promotes ERK activation in sarcoma in the context of p53 deficiency, while the effects of RanBP9 loss on c-Jun expression appear subtle and may be masked by intratumoral heterogeneity, limiting our ability to detect statistically significant differences in bulk tumor analyses.

### 3.3 Functional and proteomic characterization of *R9/p53^DHet^* MEFs

**Figure 5:**
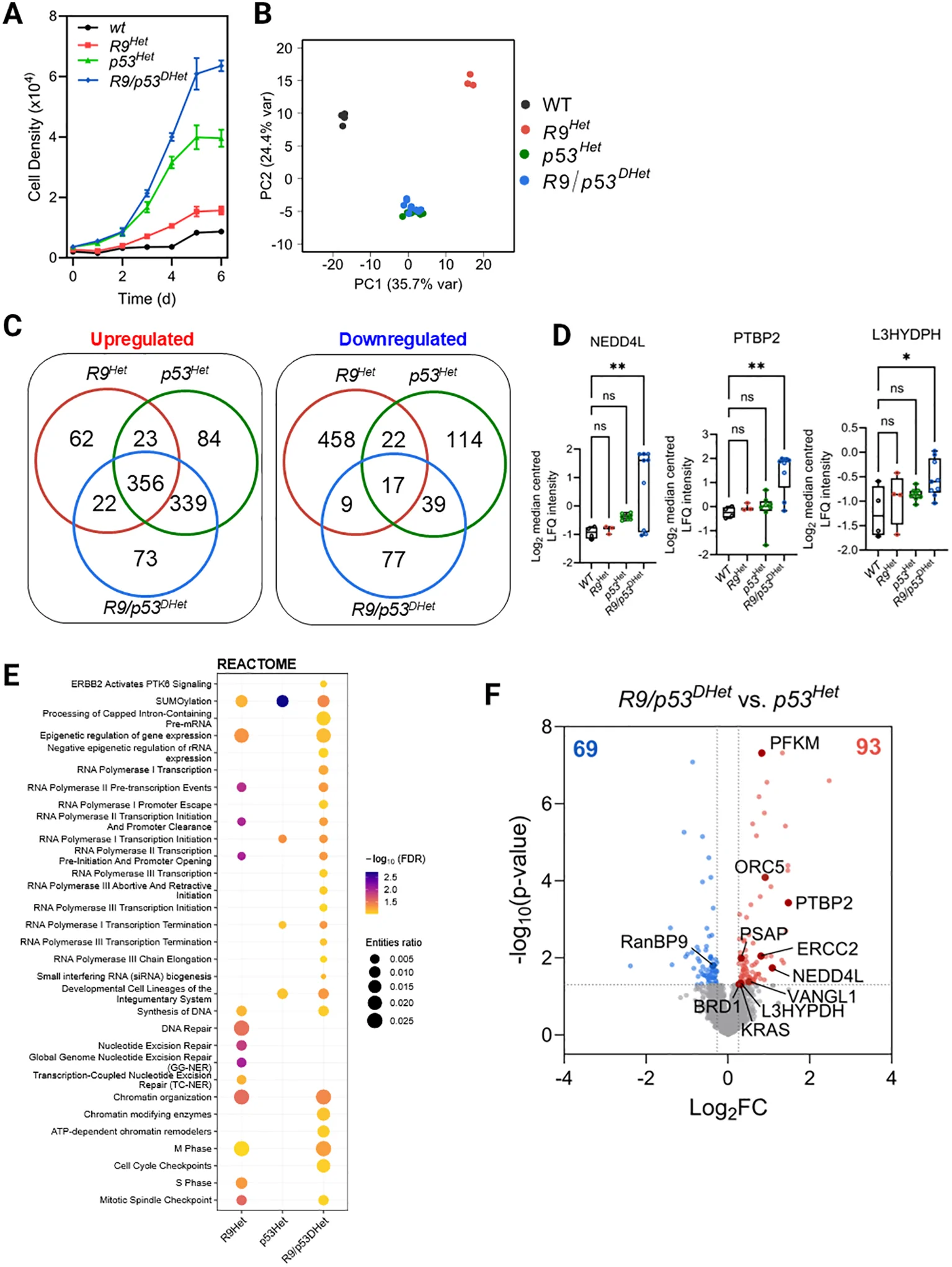
Proteomic profiling of mixed R9/p53 MEF proteomes. **(A)** Growth curves of WT, *R9^Het^*, *p53^Het^*, *R9/p53^DHet^* MEFs measured by CCK-8 assay over time. Data is presented as mean cell density ± SEM from one representative litter. **(B)** Principal component analysis of proteins that were statistically significant relative to WT proteome. **(C)** Overlap of the proteins with significant differential abundance relative to WT proteome. **(D)** Median centered intensity of NEDD4L, PTBP2, and L3HYDPH protein abundance in WT, *R9^Het^*, *p53^Het^*, and *R9/p53^DHet^* proteomes. Boxplot midline indicates median values, bounds of the box indicate 25^th^ and 75^th^ percentiles. Statistical significance was determined by one-way ANOVA with Dunnet test. *p < 0.05; **p < 0.01; ns, non-significant. **(E)** Top Reactome terms of upregulated proteins in *R9^Het^*, *p53^Het^*, *R9/p53^DHet^* relative to WT proteome. Entities ratio depicts the proportion of proteins represented in the respective Reactome pathway. **(F)** Volcano plot of proteins with log_2_ fold-change (FC) is shown on x-axis and -log_10_ (p-value) shown on y-axis.

To extend our *in vivo* findings and better understand the biological consequences of the combined heterozygous loss of *Ranbp9* and *Trp53*, we generated WT, *R9^Het^*, *p53^Het^*, and *R9/p53^DHet^* primary MEFs. These mesenchymal-lineage cells were used as a model to selectively interrogate cooperativity of *Ranbp9* and *Trp53* deletion, avoiding other genomic alterations present in most sarcoma cell lines. First, we functionally characterized *R9/p53^DHet^* MEFs. Consistent with immunohistochemical results, *R9/p53^DHet^* MEFs were more proliferative than *p53^Het^* cells (**Figure 5A**). Because the loss of RanBP9 was shown to dysregulate apoptosis and survival (30, 31, 33, 34), we hypothesized that loss of RanBP9 may further enhance pro-survival properties following p53 loss. To test this, we assessed survival properties of *R9/p53^DHet^* following DNA damage. However, we did not observe any difference in cell viability in response to either cisplatin or IR (**Supplementary Figures 5A–C**). Furthermore, the loss of RanBP9 did not impact p53 stability in response to cisplatin (**Supplementary Figures 5D, E**). Recently, the CTLH catalytic subunit MAEA was reported to regulate homologous recombination (HR) by promoting stability of replication forks following replication stress (58). Given recent evidence that the CTLH complex regulates homologous recombination (HR) (58, 59), we also assessed the sensitivity of *R9/p53^DHet^* MEFs to olaparib, a PARP inhibitor that induces synthetic lethality in cells with impaired HR-mediated DNA repair. IC_50_ analysis revealed no significant change in olaparib sensitivity across cell lines relative to control cells, suggesting that heterozygosity in either or both p53 and RanBP9 either does not significantly affect HR efficacy or their sensitivity to PARP inhibition (**Supplementary Figure 5F**). Overall, the combined loss of p53 and RanBP9 does not significantly affect MEFs cell viability upon genotoxic stress.

Second, we characterized the proteomes of WT, *R9^He^*^t^, *p53^Het^*, and *R9/p53^DHet^* MEFs under steady-state conditions by global label-free quantitative (LFQ) proteomics. On average, 6291 proteins were quantified across each group (**Supplementary Figure 6A**). Principal component analysis (PCA) was performed to assess similarity across groups using proteins with an absolute fold change greater than 1.2 and a *p*-value less than 0.05 relative to the proteome of WT cells (**Figure 5B**). Strikingly, the proteomes of *p53^Het^* and *R9/p53^DHet^* cells clustered together, suggesting high similarity. To identify differentially expressed proteins, we performed pairwise comparisons between *R9^Het^*/WT, *p53^He^*^t^/WT, and *R9/p53^DHet^*/WT proteomes (**Supplementary Figure 6B**; **Supplementary Dataset 3–5**). As expected, RanBP9 was significantly decreased in *R9^Het^* and *R9/p53^DHet^* proteomes (**Supplementary Figure 6C**). We did not observe any compensatory upregulation of RanBP10, which has been observed in other cell types following total ablation of RanBP9 (59, 60). The CTLH substrate adaptor muskelin (MKLN1), a known degradative substrate of the CTLH complex (61), was significantly increased in the *R9^Het^* and *R9/p53^DHet^* proteomes. Interestingly, this increased expression was also observed in the *p53^Het^* proteome. Overlap of significantly increased proteins revealed a considerable agreement between *p53^Het^* and *R9/p53^DHet^* cells, which is consistent with their similarity from the PCA plot (**Figure 5C**). Next, we compared differences in protein abundance across all groups. Notably, E3 ubiquitin-protein ligase NEDD4-like (NEDD4L), RNA binding protein polypyrimidine tract-binding protein 2 (PTBP2), and metabolic enzyme trans-3-hydroxy-L-proline dehydratase (L3HYPDH) were uniquely increased in *R9/p53^DHet^* proteomes (**Figure 5D**). Finally, we used the Reactome database to compare differences in biological processes associated with proteins that were differentially upregulated in the proteomes of *R9^Het^*, *p53^Het^*, and *R9/p53^DHet^* relative to the proteome of WT cells. As shown in **Figure 5E**, *R9/p53^DHet^* proteomes exhibited an enrichment in processes related to transcription, chromatin remodeling, and cell cycle (**Supplementary Dataset 6**).

Given the striking similarity between *R9/p53^DHet^* and *p53^Het^* proteomes, we performed a separate pairwise comparison between the two groups (**Figure 5F**; **Supplementary Dataset 7**). Among the 93 upregulated proteins, GTPase KRas (KRAS), bromodomain-containing protein 1 (BRD1), and prosaposin (PSAP), which are known interactors of RanBP9 (62–64), were significantly increased in the *R9/p53^DHet^* proteome. The remaining significantly increased proteins are uncharacterized with respect to their connection to the CTLH complex, these include origin recognition complex subunit 5 (ORC5), ATP-dependent 6-phosphofructokinase (PFKM), and general transcription and DNA repair factor IIH helicase subunit XPD (ERCC2). Notably, ORC5 was not detected in WT or *R9^Het^* proteomes. Furthermore, the abundance of PFKM and ERCC2 did not differ significantly when evaluated across all four groups, indicating that this difference is specific to *p53^Het^* and *R9/p53^DHet^* proteomes (**Supplementary Figure 6B**). Pathway analysis of differentially expressed proteins between *R9/p53^DHet^* and *p53^Het^* proteomes is reported in **Supplementary Figure 7**. Interestingly, RNA pol II-related transcriptional regulations were part of the top pathways represented by up-regulated proteins, whereas down-regulated proteins were split between several pathways including epigenetic regulation and vesicle-mediated transport.

### 3.4 Alterations in CTLH complex genes are observed in sarcoma patients

**Figure 6:**
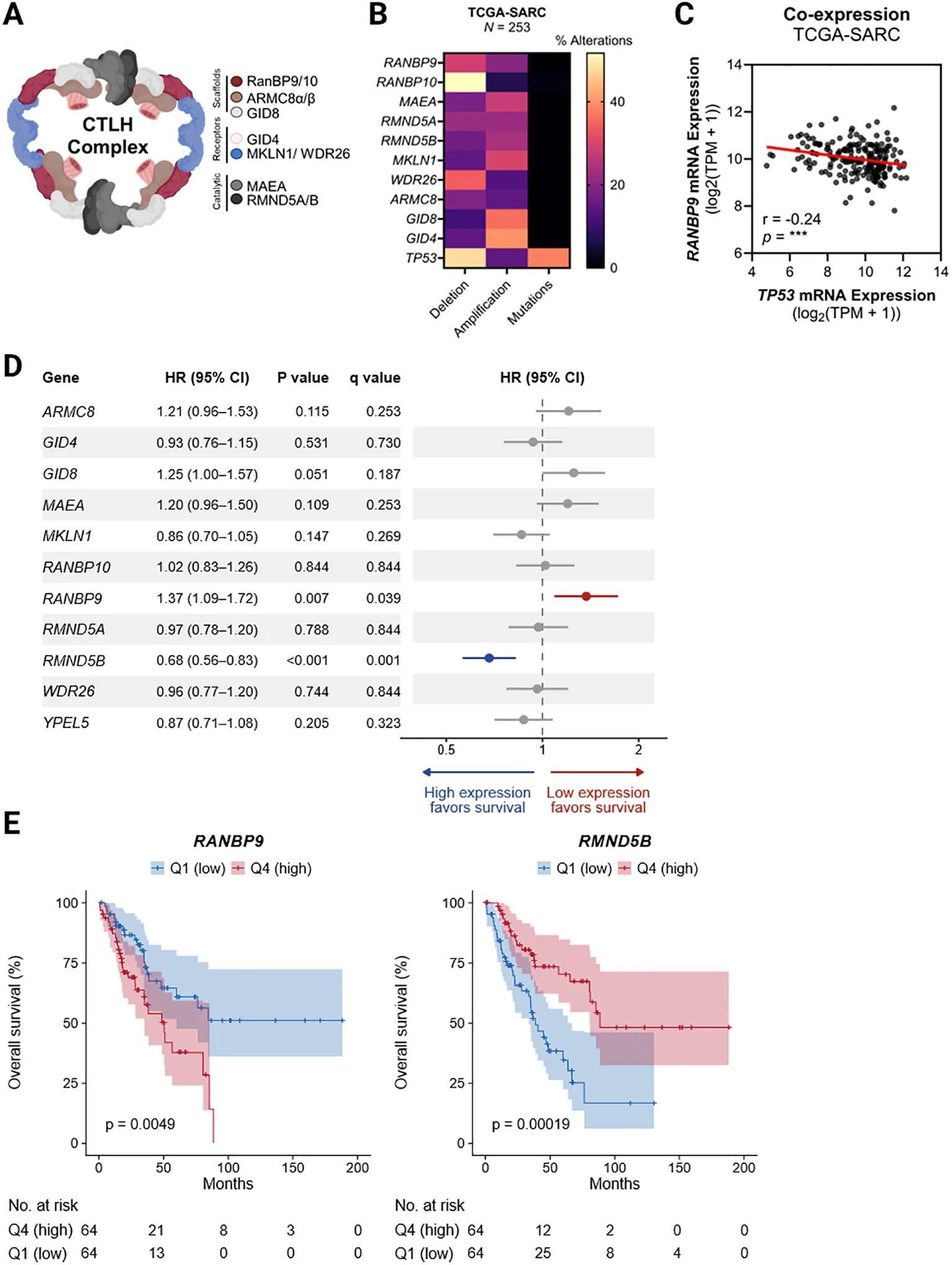
Clinical evaluation of the CTLH complex status in sarcoma. **(A)** Schematic of the CTLH complex. **(B)** Heatmap of genomic alterations in CTLH complex genes and *TP53* from TCGA-SARC (PanCancer Atlas; *n* = 253). **(C)** Co-expression analysis of *TP53* and RANBP9 mRNA. Statistical significance was determined using Pearson’s coefficient (r) with associated *p* value. **(D)** Forest plot of multivariable Cox proportional hazards analysis for CTLH subunit expression and overall survival. Expression was modelled as a continuous variable and adjusted for sex, age, and histologic subtype. Hazard ratios (HR) are shown with 95% confidence intervals (CI). *P* values were determined from the adjusted Cox model. Q-values are the corresponding p values after Benjamini-Hochberg correction. **(E)** Kaplan-Meier overall survival analysis by *RANBP9* and *RMND5B*, comparing the highest (Q_4_) and lowest (Q_1_) expression quartiles. Statistical significance was determined by log-rank test.

To determine the clinical significance of CTLH complex status in sarcoma patients, we assessed the prevalence and correlation of CTLH complex genes using the TCGA-SARC dataset (*n* = 253). Copy-number alterations (CNA) were common across CTLH genes, exhibited the highest rate of deletion events associated with *RANBP10* (**Figure 6A, B**). Mutations in CTLH genes were rare, with only *RANBP10* (*n* = 3), *RMND5A* (*n* = 1), *MKLN1* (*n* = 1), and *GID4* (*n* = 1) harboring mutations. Co-expression analysis revealed a weak inverse relationship between *TP53* and *RANBP9* mRNA expression (r = -0.24, *p* < 0.001; **Figure 6C**). Lastly, we assessed the relationship between CTLH subunit expression and overall survival, with adjustment for age, sex, and histologic subtype. Among the 11 CTLH subunit genes tested, only RANBP9 and RMND5B subunits produced a significant correlation between expression and mortality risk (**Figure 6D**). Lower *RMND5B* expression correlated with higher mortality, consistent with a tumor suppressive role (HR: 0.68, 95% CI: 0.56 – 0.83, *p* = 9.32 × 10^-5^). Unexpectedly, higher *RANBP9* expression correlated with higher mortality (HR: 1.37, 95% CI: 1.09 - 1.72; *p* = 7.11 × 10^-3^). Furthermore, Kaplan-Meier analyses revealed a significant correlation in overall survival for *RMND5B* and *RANBP9*, when expression was stratified by quartiles (**Figure 6E**). For *RMND5B*, the median overall survival for lowest expression group versus highest expression group was 54.2 months (95% CI: 48.2 to not reached) and 81 months (95% CI: 51.0 to not reached), respectively. For *RANBP9*, the median survival of the highest expression group was 50.5 months (95% CI: 35.1 to not reached), whereas median survival was not reached for lowest expression group.

## 4 Discussion

In this study, we investigated the functional implications of *Ranbp9* loss on tumorigenesis in mice. We found that heterozygous codeletion of *Ranbp9* and *Trp53* reduced tumor-free survival and increased soft tissue sarcoma development. Furthermore, sarcomas from *R9/p53^DHet^* mice were highly proliferative and exhibited increased ERK activation. Collectively, our findings support a tumor suppressive role for RanBP9 repressing sarcomagenesis. To our knowledge, this is the first study providing *in vivo* evidence implicating RanBP9 deficiency in soft tissue sarcoma development.

Biallelic loss of *Ranbp9* alone failed to induce tumors, suggesting that CTLH inactivation is not an initiating event. Furthermore, the additional defects conferred by RanBP9 loss in *R9/p53^DKO^* mice were modest relative to *p53*^KO^ mice, likely because the strong tumor-promoting events driven by p53 loss masked any additive impact brought by RanBP9 loss. Compensatory mechanisms could also explain the lack of tumor formation in *R9*^KO^ mice. RanBP10 shares high structural conservation with RanBP9, except for an extended 109 residue N-terminal region rich in polyglutamine and polyproline stretches (8). Although RanBP9 is more abundantly expressed, studies have shown a strong inverse relationship between RanBP9 and RanBP10 expression (11, 29), suggesting compensatory regulation between the two paralogs (65). Depletion of RanBP9 can also promote RanBP10 assembly into the CTLH complex, forming functional RanBP10-CTLH complexes (60). Therefore, RanBP10 expression may preserve residual CTLH complex activity in our mouse model. This is supported by previous studies that have shown that combined deletion of RanBP9 and RanBP10 was required to fully disrupt CTLH complex regulation (19, 29). Despite their structural similarity, RanBP9-CTLH and RanBP10-CTLH complexes appear to have distinct biological roles. Complete loss of RanBP9 expression disrupts erythropoiesis and gametogenesis and results in growth retardation and sterility, whereas RanBP10 loss does not (43, 60, 66, 67). In support of this, overexpression of RanBP9 and RanBP10 results in distinct proteomic and ubiquitination profiles in non-small cell lung cancer (29). From the perspective of our proteomic data, single-copy loss of RanBP9 did not upregulate RanBP10, which suggests that complete loss of RanBP9 is required for compensatory upregulation.

We found that ERK activation was enhanced in sarcomas in a RanBP9-dependent manner, consistent with the CTLH complex’s role in negatively regulating the ERK pathway (32, 41, 57, 68). These findings support a model in which loss of CTLH activity increases ERK signaling to promote proliferation, although it remains to be tested whether ERK signaling is functionally required for sarcomagenesis. Elevated ERK signaling is a key molecular signature of UPS subtypes (69). Clinically, ERK inhibition is an ongoing therapeutic strategy, with multiple clinical trials evaluating the efficacy of sorafenib and other ERK pathway inhibitors for the treatment of soft tissue sarcomas (70). *JUN* is a recognized driver of sarcomas and is often amplified or overexpressed (71). We assessed c-Jun expression in sarcoma tissues derived from our mice and observed a trend towards increased c-Jun levels in *R9/p53^DHet^* mice; however, this increase did not reach statistical significance. This suggests that, while complete RanBP9 loss results in detectable c-Jun dysregulation in MEFs, the effects of RanBP9 dysregulation on c-Jun expression in heterozygous mice are subtle and fall below the sensitivity of our current analyses. Smaller changes in c-Jun expression in heterozygous mice may nevertheless contribute to RanBP9 loss-driven sarcomagenesis, despite not reaching statistical significance in our assays. Overall, our data suggest that c-Jun is regulated by the RanBP9/CTLH complex through a combination of direct and indirect mechanisms, acting at both the transcriptional and post-translational levels. Regulation of c-Jun is inherently complex, as its expression and stability are controlled by positive autoregulation by its own protein product (72), translational mechanisms (73, 74), and numerous signaling cascades including JNK signaling (75). Therefore, it remains challenging to define the precise contribution of the RanBP9/CTLH complex to this regulatory network.

Inactivation of *TP53* is one of the most common events in sarcomas and contributes to development through genomic instability leading to defects in multiple regulatory networks (76). Given the role of the CTLH complex in HR and transcriptional regulation (57, 58), it is tempting to speculate that the additive loss of CTLH activity may further contribute to genomic instability to promote malignant transformation. Surprisingly, we did not detect substantial reduction in cell viability following olaparib treatment in *R9/p53^DHet^* MEFs. Given that our assay detected a reduction in HR deficiency in Ranbp9-null MEFs, *Ranbp9* heterozygosity may only result in weak HR deficiency, which our assay was not sensitive enough to detect. Furthermore, sensitivity to replication stress may be masked by the heterozygous *Trp53* background as p53-deficient cells are known to bypass replication stress through the ATR-CHK1 signaling axis (77).

Our proteomic screen revealed enrichment in proteins involved in transcription and cell cycle. Consistent with cell cycle and DNA replication, ORC5, a subunit of the origin of replication complex (ORC), was upregulated in the proteome of *R9/p53^DHet^* MEFs. During late mitosis and early G_1_ phases, the ORC binds to replication origins and recruits additional factors to initiate DNA replication (78–80). Hyperactivation of replication origins is thought to contribute to genome instability in cancer cells, with overexpression of ORC subunits prevalent in many cancers including hepatocellular carcinoma and lung adenocarcinoma (81–85). Specifically, overexpression of ORC5 was shown to promote aberrant chromatin decondensation in osteosarcoma cell lines (86). Interestingly, previous work detected ORC5 as a proximal interactor of CTLH substrate adaptor GID4 (87). ORC5 contains a Pro/N-degron motif, which suggests that ORC5 could be a GID4 substrate potentially regulated to influence DNA replication.

Other promising candidates include metabolic enzyme L3HYPDH and glycolytic enzyme PFKM. Similar to ORC5, L3HYPDH was also detected as a proximal interactor of GID4 (87), though it lacks a canonical GID4 degron. L3HYPDH was enriched in *MAEA^KO^* HEK293 proteomes (12); however, the biological consequence remains unknown. We also identified PFKM as a proximal interactor of muskelin (unpublished data). Previous diGLY proteomic screens identified a reduction in PFKM ubiquitination at several sites including K372 in RanBP9 deficient HeLa cell lines (17, 39). We did not see any evidence of elevated PFKM protein levels across previous proteomic screens, suggesting that the CTLH complex may regulate non-proteolytic outcomes on PFKM. Notably, the CTLH complex regulates other glycolytic enzymes through non-degradative mechanisms (39). Future work will be needed to functionally validate these candidates and to determine whether their dysregulation contributes to sarcomagenesis.

Another area that remains to be further investigated is the role of the CTLH complex during mesenchymal differentiation. Sarcomas are thought to originate from defective terminal differentiation of mesenchymal stem cells (MSCs) or from direct transformation of primitive MSCs (88). We found reduced differentiation features in sarcomas derived from *R9/p53^DHet^* mice, suggesting that RanBP9 loss could play a role in regulating differentiation. Previous studies identified a critical role for the CTLH complex in erythroid differentiation (60). Future work characterizing CTLH complex function during MSC differentiation may help clarify how CTLH inactivation contributes to sarcoma development.

Lastly, we tested several DEGs identified from hepatic RNA-sequencing but were unable to detect a significant difference in MEF cells (data not shown), suggesting differences in tissue biology. TCGA data revealed opposite trends with expression and overall survival for RanBP9 and RMND5B in patients with sarcoma. Genomic data regarding CTLH subunits must be cautiously interpreted as mRNA and protein expression are poorly correlated for most CTLH subunits (8). Also, the significance of individual CTLH subunit overexpression remains difficult to interpret because it is unclear whether increased expression leads to the formation of additional active CTLH complexes. Confirmatory immunohistochemical analyses of CTLH protein expression across different sarcoma subtypes will be essential to validate and extend our findings. However, such profiling is currently infeasible due to the lack of reliable antibodies compatible with immunohistochemical detection.

In summary, our study provides *in vivo* evidence for CTLH complex involvement in sarcomas by demonstrating that heterozygous loss of *Ranbp9* in *Trp53* deficient mice increases tumor penetrance with a bias towards soft tissue sarcomas. RanBP9 loss likely promotes sarcoma development through the cumulative dysregulation of multiple cellular processes and signaling pathways. Our data confirmed the ERK pathway as a primary pro-tumorigenic pathway deregulated by the loss of RanBP9 and identified several additional pathways and candidate targets for further investigation. Future studies characterizing CTLH E3 ligase-p53 function using sarcoma-based models will provide further molecular insight. Overall, our findings establish RanBP9 as a candidate tumor suppressor cooperating with p53 in mesenchymal cancers.

## Data Availability

The RNA-seq processed reads data have been deposited in NCBI's Gene Expression Omnibus (90) and are accessible through GEO Series accession number GSE310308. The mass spectrometry proteomics data have been deposited to the ProteomeXchange Consortium via the PRIDE (91) partner repository with the dataset identifier PXD081521.
