# Supplementary material for: Loss of RanBP9 cooperates with p53 deficiency to promote sarcomagenesis
Source: Front Oncol. 2026 Sep 3;16:1743582. doi: 10.3389/fonc.2026.1743582 (PMC13581892; doi:10.3389/fonc.2026.1743582)
Supplement: Supplementary file 1 [file SupplementaryFile1.docx]

Supplementary Material

Gonga-Cavé et al.,

# 1 Supplementary Figures and Tables

## 1.1 Supplementary Figures

**Supplementary Figure 1: Liver senescence analysis of Ranbp9 knockout mouse kidneys and liver.** β-galactosidase assays performed on whole livers and kidneys derived from 4.5-month-old male *Ranbp9* knockout (KO) and wild-type (WT) C57BL/6 x B6129F1 mice. Liver and kidneys were fixed and stained with 1 mg/mL X-gal at 37°C and pictures were taken in 30-minute (min) increments. Picture brightness and contrast were normalized based on background. Representative image from 3 independent replicates.

**Supplementary Figure 2: Cluster heatmap analysis of gene expressions between wild-type (WT) and *Ranbp9* knockout (KO) mouse livers.** Hierarchical clustering of differentially expressed genes between WT and KO mouse liver is displayed.

**Supplementary Figure 3**: **Investigation of c-Jun expression in CTLH deficient HeLa cells**.

(***A*-*E***) Western blot analysis of c-Jun expression in selected CTLH deficient HeLa cells. Vinculin served as a loading control. Data is presented as mean ± SEM (*n* > 3). Statistical significance was determined by unpaired t-test.

(***F***) Western blot analysis of c-Jun expression following cycloheximide (CHX) treatment in WT and *MAEA^KO^* HeLa cells. Vinculin served as a loading control. Data is presented as mean ± SEM (*n* = 3). MAEA was assessed on a different western blot with the same samples.

**Supplementary Figure 4: Combined heterozygous loss of *Trp53* and *Ranbp9* does not significantly impact nuclear features and pleomorphism in sarcomas.** Histological grading of (***A, D***) primary cell lineage, (***B, E****)* nuclear features, and (***C, F***) pleomorphism of sarcoma sections. Quantification of index grade is shown as mean ± SEM. Statistical significance was determined by unpaired t-test with Welch’s correction. ns = non-significant.

*Legend on the next page*

**Supplementary Figure 5: DNA damage sensitivity and survival response in *R9/p53^DHet^* MEFs**

(***A***) Cell viability of mixed *R9/p53* MEFs treated with 10 µM cisplatin, measured by CCK-8 assay over time. Data is presented as mean ± SEM cell viability.

(***B***) Cell viability of mixed *R9/p53* MEFs exposed to 4 Gy of ionizing radiation, measured by CCK-8 assay after 96 h. Quantification of cell viability is shown as mean ± SEM (*n* = 4). **p* < 0.05; ns = non-significant.

(***C****)* Clonogenic survival of mixed *R9/p53* MEFs following ionizing radiation (IR). Colonies were stained with crystal violet after 15 days. Representative images (*left*) and quantification of plating efficiency and survival fraction (*right*) is shown as mean ± SEM (*n* = 4). Statistical significance was determined by one-way ANOVA with post-hoc Turkey test. ***p* < 0.01; ns = non-significant

(**D**) Western blot analysis of p53 activation in *WT* and *R9^KO^* MEFs treated with 10 µM cisplatin for 24 h. Vinculin served as a loading control. Quantification of fold change is shown as mean ± SEM. Statistical significance was determined by unpaired t-test (*n* = 4). ns = non-significant.

*(****D****)* Western blot analysis of mixed *R9/p53* MEFs treated with 10 µM cisplatin for 24 h. Vinculin served as a loading control. Quantification of fold change is shown (mean fold change ± SEM, *n* = 4). Statistical significance was determined by unpaired t-test. ns = non-significant.

(***F***) Olaparib sensitivity assay of mixed *R9/p53* MEFs treated with logarithmic concentrations of Olaparib or DMSO for 72 h. Cell viability was measured using CCK-8 assay. IC_50_ for each group is reported in table.

*Legend on the next page*

**Supplementary Figure 6:	Extended proteomic profiling of mixed R9/p53 MEF proteomes**

(***A***) Number of quantified proteins across WT (*n* = 4), *R9^Het^* (*n* = 3), *p53^Het^* (*n* = 8), and *R9/p53^DHet^* (*n* = 9) MEF proteomes using label-free quantification (LFQ).

(***B***) Volcano plot of all proteins with log_2_ fold-change (FC) is shown on x-axis and -log_10_(p-value) shown on y-axis.

(***C***) Median centered intensity of indicated proteins across WT, *R9^Het^*, *p53^Het^*, and *R9/p53^DHet^* proteomes. Boxplot midline indicates median values, bounds of the box indicate 25^th^ and 75^th^ percentiles. Statistical significance was determined by one-way ANOVA with Dunnet test. ***p < 0.001; ****p < 0.0001; ns = non-significant. Number of quantified proteins across WT (*n* = 4), *R9^Het^* (*n* = 3), *p53^Het^* (*n* = 8), and *R9/p53^DHet^* (*n* = 9) MEFs using label-free quantification (LFQ).

**Supplementary Figure 7: Reactome pathway analysis of differentially expressed proteins.**

Top Reactome terms of differentially expressed proteins in *R9/p53^DHet^* relative to *p53^Het^* proteome samples. Entities ratio depicts the proportion of proteins represented in the respective Reactome pathway.

## 1.2 Supplementary Tables

| **Supplementary Table 1. Primers for genotyping** | | |
| --- | --- | --- |
| **Gene** | **Target** | **Primer Sequence** |
| *Ranbp9* | Vr_3_ | 5′-GGC TAC CGG CTA AAA CTT GA-3′ |
|  | WT_a_ | 5′-TAC CAT GAC CTG CCC GTG A-3′ |
|  | WT_s_ | 5′-CTT TAA TTT GGG GAC CAG AG-3′ |
| *Trp53* | WT F | 5′-GTA TCT GGA AGA CAG GCA GAC-3′ |
|  | WT R | 5′-TGT ACT TGT AGT GGA TGG TGG-3′ |
|  | KO F | 5′-TAT ACT CAG AGC CGG CCT-3′ |
|  | KO R | 5′-TTC CTC GTG CTT TAC GGT ATC-3′ |

| **Supplementary Table 2. PCR conditions for Ranbp9 and Trp53 genotyping** | | | |
| --- | --- | --- | --- |
| **Gene** | **Target** | **PCR Conditions** | |
| *Ranbp9* | WT  KO | 1. 94 °C, 3:00 2. 94 °C, 0:45 3. 60 °C, 0:45 4. 72 °C, 1:00 | 5. GOTO step 2, 34X 6. 72°C, 10:00 7. 4°C, ∞ |
| *Trp53* | WT | 1. 94 °C, 3:00 2. 94 °C, 0:45 3. 60 °C, 0:45 4. 72 °C, 1:00 | 5. GOTO step 2, 34X 6. 72°C, 10:00 7. 4°C, ∞ |
|  | KO | 1. 94 °C, 3:00 2. 94 °C, 0:45 3. 67 °C, 0:45 4. 72 °C, 1:00 | 5. GOTO step 2, 34X 6. 72°C, 10:00 7. 4°C, ∞ |

| **Supplementary Table 3. Western blot antibodies used in this study** | | | |
| --- | --- | --- | --- |
| **Antibodies** | **Dilution** | **Supplier** | **Identifier** |
| **Primary** |  |  |  |
| Actin | 1:1000 | Sigma-Aldrich | A5441 |
| ARMC8 | 1:250 | Santa Cruz Biotechnology | E-1; sc-365307 |
| c-Jun | 1:500 | Cell Signaling Technology | 9165S |
| GID4 | 1:1000 | Novus | NBP1-53185 |
| MAEA | 1:1000 | Proteintech | 15238-1-AP |
| MKLN1 | 1:500 | Santa Cruz Biotechnology | C-12; sc-398958 |
| p53 | 1:500 | Cell Signaling Technology | 2524S |
| RanBP9 | 1:2000 | Bio Academia | 71-001 |
| RMND5A | 1:2000 | Yenzyme Antibodies | Custom |
| WDR26 | 1:2000 | Abcam | ab85962 |
| Vinculin | 1:10000 | Cell Signaling Technology | 13901S |
| **Secondary** |  |  |  |
| IRDye680RD | 1:5000 | LI-COR Biotechnology | 926-68072 |
| IRDye 800CW | 1:5000 | LI-COR Biotechnology | 2926-32213 |

| **Supplementary Table 4.** **Characteristics of the mouse cohorts used in this study** | | | | | | |
| --- | --- | --- | --- | --- | --- | --- |
| **Characteristic** | **WT**  **(n = 12)** | ***R9^KO^***  **(n = 29)** | ***p53^Het^***  **(n = 18)** | ***R9/p53^DHet^***  **(n = 22)** | ***p53^KO^***  **(n = 6)** | ***R9/ p53^DKO^***  **(n = 10)** |
| Sex – no. (%) |  |  |  |  |  |  |
| Male | 2 (17) | 15 (52) | 8 (44) | 8 (36) | 4 (66) | 8 (80) |
| Female | 10 (83) | 14 (48) | 10 (56) | 14 (64) | 2 (33) | 2 (20) |
| Tumor development – no. (%) | 0 (0) | 0 (0) | 5 (28) | 12 (54) | 6 (100) | 5 (100) |
| Male | 0 (0) | 0 (0) | 2 (11) | 1 (4) | 4 (67) | 3 (60) |
| Female | 0 (0) | 0 (0) | 3 (17) | 11 (50) | 2 (33) | 2 (40) |
| Pre-weaning mortality – no. (%) | 0 (0) | 0 (0) | 0 (0) | 0 (0) | 0 (0) | 3 (30) |
| Dermatitis | 1 (8) | 7 (24) | 0 (0) | 3 (14) | 0 (0) | 2 (20) |
| Malocclusions | 1 (8) | 3 (10) | 0 (0) | 0 (0) | 0 (0) | 2 (20) |
| Other comorbidities* | 0 (0) | 3 (10) | 0 (0) | 0 (0) | 0 (0) | 1 (10) |

*Other comorbidities include self-mutilation due to excessive licking, and penile and rectal prolapses.

**1.3	Excel Datasets**

**Supplementary Dataset 1:** Differentially expressed genes identified by hepatic RNA sequecing analysis of *Ranbp9* knockout mice compared with wild-type controls.

**Supplementary Dataset 2:** Full unfiltered Reactome and GO pathway analysis of upregulated DEG from hepatic RNA-seq analysis of *Ranbp9* knockout mice compared with wild-type controls.

**Supplementary Dataset 3:** Full unfiltered differentially expressed genes identified by mass-spectrometry analysis of *Ranbp9* heterozygous (*R9^Het^*) mice compared with wild-type controls.

**Supplementary Dataset 4:** Full unfiltered differentially expressed genes identified by mass-spectrometry analysis of *Trp53* heterozygous (*p53^Het^*) mice compared with wild-type controls.

**Supplementary Dataset 5:** Full unfiltered Differentially expressed genes identified by mass-spectrometry analysis of double heterozygous *Ranbp9* and *Trp53* (*R9/p53^DHet^*) mice compared with wild-type controls.

**Supplementary Dataset 6:** Full unfiltered reactome pathway analysis of differentially expressed proteins across MEFs.

**Supplementary Dataset 7:** Full unfiltered differentially expressed genes identified by mass-spectrometry analysis of double heterozygous *Ranbp9* and *Trp53* (*R9/p53^DHet^*) mice compared to *Trp53* heterozygous (*p53^Het^*) mice.

**Supplementary Dataset 8:** Full unfiltered reactome pathway analysis of differentially expressed proteins found in double heterozygous *Ranbp9* and *Trp53* (*R9/p53^DHet^*) mice versus *Trp53* heterozygous (*p53^Het^*) mice.

There are issues with the following figures: Fig 1: Panel C and D text are blurry. Fig 5: Panel E text is blurry.We do not know why this happened as our original figure is fine.
